# The New Serum Treatment for Epilepsy

**Published:** 1915-06

**Authors:** William Held

**Affiliations:** Chicago, Ill.


### The New Serum Treatment For Epilepsy

By WILLIAM HELD, M. D., Chicago, Ill.

When discussing the treatment of epilepsy our first thought is directed toward the large, the formidable array of remedies which have been lauded, abused and discarded in the treatment of this malady. Thinking of epilepsy, the general practitioner has in mind the awful chronic disease, considered incurable, the occurence of which in his practice, makes it incumbent upon him to disclose to the interested parties that his medical skill has encountered a foe which he cannot hope to successfully cope with.

When looking back over the remedies once applied in the treatment of epilepsy, and their number is legion, we encounter through the misty past the sorcerer in all earnestness and with a self assertion, befitting a worthier cause, going through his antics in an attempt to ''cast out'' the demon from the epileptic, the possessed one.

Truth compels the admission, that as far as the epileptic is concerned, the fervor of the holy medicine man of the dark ages, whose treatment consisted in banishing the demon from the patients body by the use of red hot irons, has done as little good as has the modern M. D. who followed in the wake of the ancient faith healer, with his armament of inefficient and often harmful remedies. In the face of the fact that all medication up to this time has not sufficed to produce an anti-epileptic remedy combining efficiency plus harmlessness in view of the fact that many once enthusiastically heralded remedies have failed to justify themselves and above all, the tragic spectacle that despite all existing anti-epileptic remedies our insane asylums, our infirmaries, our homes for feeble minded and our epileptic colonies are still brimming over with mentally degenerated epileptics, in view of all this, it is with pardonable reluctancy that one approaches with something new about so old a malady as epilepsy. At first thought it would seem that where so much had been tried, nothing new could be found; man's ingenuity to be exhausted and that epilepsy will continue to baffle medical science as it has done from time immemorial.

We have all been taught to look upon epilepsy as a lesion of the brain cortex and into the hands of all of us, metamaphorically speaking, have the apostles of such teachings pressed a good sized club with which to knock epilepsy into temporary abeyance whenever this enemy raises its head. The name of this weapon as all know, is bromide, the accepted routine treatment for epilepsy.

Se we have indeed upon our errands of ministration to the sick met the dreaded foe and dealt a stunning blow with the chief anti-epileptic sedative.

Striking thus at epilepsy we were unable to protect the patient from part of the stunning force inflicting injury upon him too.

Statistics and authoritative opinion support my claim, that the use of bromide has permanently deprived many epileptics of what sound mentality the disease itself has left them, has sped the way of many such patients over the border line that separates constitutional disease from insanity. Those who incline to look upon this statement as an exaggeration, should look to the multitude of bromidized insane, incompetent, degenerated, homocidal, perverted epileptics who abound in institutions and private homes everywhere. After having seen enough of these unfortunates let them compare them with the few epileptics, whose good luck, amidst their misfortune, it was to be under the guidance of people who stuotly refused to bromidize their charge.

If bromide could be administered for long periods, thereby arresting the epileptic attacks, keeping the patient free from these dreaded seizures, without subduing cerebration and without producing the gastric and other disturbances so common during bromide treatment; if a method of bromide treatment had been evolved under which the patient remains free from attacks and at the same time maintains an unclouded mind, then there would be less urgent need for a remedy to supplant bromide in its spell suppressing effect. When a remedy is found that will reduce the number and severity of seizures, arrest attacks and gradually increase the length of spell free intervals, and do all this without in any manner impairing the mental or physical condition of the patient, then we may assume to have entered the gateway which will ultimately lead to complete recovery from epilepsy.

A new therapeusis, the production of an anti-epileptic serum, could naturally follow only in the wake of a new pathology of epilepsy. It became evident that the present day views of ethiology and pathology of epilepsy were untenable, that present day theories do not harmonize with certain phenomena as elicited in carefully selected and treated cases with the new serum.

The intestinal flora furnishes a very important source of supply for the converting of septic material into toxins. Epileptogenic toxins are thus manufactured and absorbed into the blood stream where they accumulate. In turn toxins circulate through the system, poison the brain centers and under symptoms of toxicity, auto-intoxication, give rise to confusion, mental derangement, paralysis, convulsions, epilepsy.

Doctors have tabooed various foods and formulated a restricted diet, the gist of which dietary is the recognition that something in the epileptics economy possesses the power to manufacture these foods into products favorable to the release of epileptic seizures. Diet the patient as we may there always seems active some agency, drawing from whatever available food and waste material of the epileptics system sufficient epileptogenic toxins, absorbing the same into the blood stream, bathing and saturating every cell of the body and registering its presence by the release of epileptic seizures. That these toxins are produced from foods and sources which do not affect the non-epileptic, strongly hints to a vast difference between the mechanism of the non-epileptic and epileptic metabolism. The converting of body material into epileptogenic toxins, neurotoxic material is due to the pathological metabolism of the epileptic, the epileptic metabolism. Something must be done to arrest or blight this epileptogenic function, some agency introduced into the patients economy which will interfere with, counteract or destroy this pathological action.

As long as these toxins are manufactured, they will accumulate until sufficient to irritate the cortex into epileptic explosions. Depending upon the degrees of the amount of toxins present, but not less so upon the state of cortical resistance, the toxic influence will be light or severe, the attacks ranging from a slight, fleeting confusion of a few seconds duration to the most violent and severe attacks of status epilepticus, accompanied by the voidance of feces, and urine and not infrequently terminating in the patients death.

Could we introduce some element into the blood stream, in the presence of which the epileptogenic function could not assert itself, in the presence of which the SPECIFIC SELECTIVE FUNCTION OF THE BRAIN CORTEX were subdued, then we are justified in taking courage at the prospect. Such agency should attack the ultramicroscopic organism or whatever is the responsible element in the epileptics blood; it should by the blood stream be carried to the brain and bathe the cortical cells in it for the purpose of **neutralizing the cortical cells function, of uniting with epileptogenic toxins such ferments as result in epileptic attacks.** The element introduced must be one that does in no way interfere with normal cerebration, it must not stun, not anaesthetize the brains sensibility, for if it does that, it is no longer a curative step at all but merely a disguise for a condition still remaining. Such a substance would have the office of charging the patients blood with constituents, in the presence of which the epileptic attractive principle could not function, it would weaken or render powerless these forces.

The cortex cells secrete a specific, characteristic substance, a ferment not generated by any other organ. These ferments exert a specific affinity for introduced anti-toxins, consisting of similar cell substance. In the presence of such material the brain cells are inhibited in their epileptogenic function and epileptogenic ferments are neutralyzed. Continued bathing of the brain cells in properly prepared anti-epileptic substance and repeated blighting of epileptic ferments will finally result in the establishment of a balance between body fluids present and such introduced, to the end of creating what I have termed the non-epileptic habit. It has been said that every cell has a memory. Give every living cell in the epileptics' economy cause to remember that the epileptic attacks have ceased and they will actually cease.

The memory of cells must be accepted in the light of intricate biological and chemical function, a memory developed by habitual response, on the part of living cells to the stimulus exerted by certain alkaloids. It may be compared to the drug craving of a patient whose objective mind, realizing the dangers of habitual drug ingestion and in full possession of his sound mentality, desires to discontinue the use of the habit forming drug. Every drug steeped cell of his system however, cries out against the sober judgment of the mind, demanding gratification of the habit; the memory. So in epilepsy, the longer the patients brain cells are held free from epileptic toxins or ferments, the longer the spell free intervals, the deeper will the non-epileptic habit be established. All this applies of course only to spell freedom not produced by drugs.

In case of Jacksonian epilepsy, the damaged cortex presents a locus minoris resistentia hence a hypersuseptible area for epileptic toxins coursing in the patients blood and also creative of epileptogenic ferment production by the injured brain cells. The weakened brain cortex offering no resistence to the responsible substances, whether ferments or toxins, are poisoned first or perhaps alone, of all the organs and thus enable the observer to notice the registering of the toxins by release of convulsions and mental derangement. Treatment of the epileptic habit merely means to afford to the recently damaged cortex time to repair, thereby placing it upon an equal basis of resistence with the cortex of the idiopathic epileptic. Does not this point to some other force in the physiology of the epileptic, some other factor that possesses the power to record its presence upon the cortex by releasing epileptic attacks, a cause remaining and still controlling unabated after removal of the supposed cause. This too supports the contention that in the blood, perhaps in the brain (carried to it by the blood) of the epileptic, is contained an epileptogenic material, a toxin which poisons the cortical layer of cells and results in epileptic attacks. These toxins do not cause continuous seizures because the necessary toxic material is being continuously produced and accumulated and only after reaching a certain ratio to the epileptogenic principle (call it ferment) present in the blood or brain, is the charge sufficient to upset the centers.

The specific affinity which epileptogenic toxins seem to exhibit for the brain cortex have caused me to draw comparisons between epilepsy and rabies. While conclusions to which such comparison tempts may appear rather bold, the fact of a striking similarity remains. Laboratory work conducted with the mentioned similarity in mind, divest first formed conclusions of much of their apparent daring character and lend much ground to expect important developments.

In symptoms as well as in pathology of the two conditions, much similarity may be found. Rabies as well as epilepsy depend upon an unknown, respectively undemonstrated organism. It is only of later date, that Noguchi is said to have isolated the organism of rabies. Both diseases have the cortex as a selective site or nidus for the responsible toxins. In epilepsy as well as in hydrophobia, one may observe perspiration, vomiting, pain, metal depression, restlessness, insomnia, joint pain, diarrhea, constipation, dizziness, tintus aurium, dyspepsia, muscular contraction and convulsions. In hydrophobia recovery is unknown, (barring one case cited in literature) and in epilepsy the tenacious recurrence has brought that malady under the head of uncurable conditions. In both diseases the fundamental cause is a neurotoxic material (I think a ferment) in rabies the course of travel of the virus to the centers being along the nerves. In rabies too, immunizing results were obtained long before a reasonable organism could be demonstrated, so that the treatment of epilepsy by serum injection is, as was the case with rabies, based upon empiricism. Whether anti-epileptic serum influences the germ destroying phagocytic leucocytes, stimulating the same to increased activity, creating in them, by supplying epileptic nourishment, a habit to act perferably upon epileptogenic substances (either germ, virus, or ferment) or whether the serum acts as a detoxicator after having reached the cortex, is not determined. Experiments and clinical results demonstrate that repeated inoculation with anti-epileptic serum establishes freedom from attacks, in a gradually increasing length of spell freedom. This is true of the animal as well as of the human being.

The protective, preventive, detoxicating and toxin blighting properties may reasonably be assumed to be inherent in a serum developed from epileptic's blood, laden with epileptogenic substances. This view is materially strengthened by observations to the effect that epileptic toxins automatically produce an anti-toxin under certain conditions, in the patients body. (Auto-genous). We find that epilepsy at times ceases as if of its own accord, spontaneously. Anti-toxins thus formed appear to protect the system for a period lasting until the original cause re-asserts itself. This event is followed by the production of new toxins, in excess of the prophylactic dose of the auto-genous anti-toxin. We all know of cases where epilepsy, after having defied all medication for years, has suddenly ceased, leaving the patient free from attacks for a time, then to re-appear. In such instances one is justified in accepting the theory that the epileptic principle of the blood was effectively blighted or counteracted, the pathological metabolism controlled by a sufficient dose of auto-genous anti-toxin.

The anti-epileptic serum, no doubt, was gradually generated as a result of epileptic seizures and set free by some unknown mechanism of metabolism.

### Overcoming Disadvantages.

In over-coming the disadvantages which were part of the early serum injections such as excessive local irritation and unpleasant toxic reaction, the fact was emphasized that slight changes in composition and mode of procedure are often followed by remarkable and decided alteration of the physiological effects of the so altered product. So for instance, I have found that the anti-rabific virus, used alone, has no antiepileptic property; epileptic serum alone often possesses highly unpleasant and irritating features at the same time lacking many of the characteristics pertinent to the attainment of the desired effect. Cerbo spinal fluid, unmixed, also proved ineffectual while a combination of any of the mentioned substances left discouragingly much to be desired.

Considerable experimentation and long series of tests finally led to the production of anti-epileptic serum, which has its foundation in a method by which all three of the named substances are employed. The anti-epileptic serum gained by my process possesses a minimum of irritating power and has in actual practice proven effective to arrest epileptic attacks entirely replacing bromide. The present method of preparing this serum is a tedious one, commercially not tempting, and allows of great improvement, which eventually will be obtained. The results thus far achieved justify the expenditure of much time and patience, as is amply demonstrated by the fact that confirmed epileptics have been kept free from attacks for periods ranging from eight weeks to nine months by no other treatment but the injection of the serum plus administration of intestinal antiseptics. Attacks occuring during serum treatment were characterized by an unusual mildness and the absence of post-epileptic stupor. Physicians who have treated patients by injections of anti-epileptic serum, report a decided improvement in the mental conditions of their patients. Clinical observations have demonstrated that anti-epileptic serum so prepared is not only fully able to replace bromides as far as the arrest, respectively prevention of the seizures is concerned, but that it also lacks the well-known disastrous drawbacks which attach to prolonged bromide administration. Moreover, considering the mental state of the patient, anti-epileptic serum strongly tends to establish a very noticeable improvement with a tendency toward freedom from attacks, while bromide diminishes this chance in the ratio at which the patients mental faculties deteriorate by the bromide medication. The state of brain fog and sluggishness so often seen in bromidized epileptics is never experienced with the serum treatment. A balance between the epileptic attractive blood constituents and the newly introduced protective elements seems to be established, which finally should be maintained without further introduction of new serum.

The entire subject up to date, may be summed up in the following conclusions: 1. That epilepsy is a toxemia depending upon the presence of epileptogenic toxins, manufactured from products present or suddenly thrown on the system of the patient. The intestinal flora furnishing a major part of the supply. 2. That the epileptics metabolism is pathological in as much as it converts food and waste products into epileptogenic material instead of disposing of such in the normal manner incidental to a healthy metabolism. The prime factor in the perverted metabolism of the epileptic seems to be a peculiar characteristic of the blood to absorb toxic material into the blood stream and retain the same as a neurotoxic poison. This characteristic should be called "epileptogenic principle." This principle prevents the normal function of leucocytes, which would absorb, devour and destroy fetid matter. 3. Epileptogenic toxins irritate every tissue in the body but have in the brain cortex a nidus of special favorable condition whereupon to register their presence by the release of epileptic seizures. 5. Jacksonian epilepsy means that the brain cortex in consequence of injury has been reduced in its resisting power and is thereafter attacked by epileptogenic products, which are independent of the injury itself.

It is my personal opinion or rather deeply rooted conviction, that continuous research will ultimately result in the discovery of a positive specific against epilepsy and I most strongly feel that this goal will be achieved by following the course outlined and pursued by my present experiments in that field. I would warn against construing anything I have said as a claim that I have discovered a cure for epilepsy and impress the necessity from abstaining to spread such erroneous news, so that in the light of a future, vaster knowledge no rude awakening may be ours. I do however feel justified at this time, based upon the experience of many physicians, to urge discontinuance of bromide and supplant this drug by the use of anti-epileptic serum in order to prevent the mental decay of the epileptic.

5511 Higgins Avenue.

---

**Crenation of Red Blood Cells—Significance.** Robert L. Watkins of N. Y., Am. Med., March, 1915, considers that these are due to the partial disruption of the red cell into its constituents, the microzymes and that, if crenation is especially marked, as in neurotic and alcoholic cases, it indicates a weakened resistance acting through the nervous system. He reasons that the relative lack of crenation in tuberculosis depends on the fact that the nervous force is not particularly reduced. (Note: Without disputing the microzym theory, it is difficult to understand how a nervous influence can act on the red cells, except through some definite failure of nutrition. Nor do we think that the roundness of crenations supports the theory as, under ordinary physical laws, any analogous separation into parts would follow this shape).

---

**Biologic Classification of Pneumococci.** Frederick M. Haynes, Richmond, Old Dominion Jour., Mch. 1915. Pure cultures of pneumococci can usually be obtained by injecting mixed infectious material into white mice, which die of pure pneumococcus septicaemia. Strains of pneumococci cannot be recognized by direct staining or cultural methods but can be separated by biologic methods. These strains have very different virulence. Group I of Cole and Dochez was found in 34 patients, of whom 8 died (24%); Group II in 13 patients of whom 8 died (61%); P. mucosus in 10 patients of whom 6 died (60%); Group IV (heterogeneous) in 15 patients of whom 1 died (7%). He condemns shot-gun mixtures of serums and states that one large dose of active specific immune serum, intravenously, is sufficient absolutely to sterilize the blood.

---

**Urinary Test for Syphilis.** Charles H. Walker and Frederick Klein of N. Y. Am. Med., Apr. 1915. A morning sample is used, cleared of albumin, phosphates and sediment if necessary. 6 C.C. are shaken thoroughly with 1% of iodin in carbon tetrachlorid, the urine being first acidulated with acetic or phosphoric acid if alkaline. If active syphilis is present, the lower layer becomes pink or purple-red. A control is made. If mercury, arsenic, salvarsan, etc., have been used and a negative test occurs, 3-4 C.C. of decinormal iodine solution U. S. P. are added to the first comparative test in one tube and ½-1 C.C. of 10% phosphoric acid in another. If both tests still result negatively, syphilis may be excluded.

